# Iron – a background article for the Nordic Nutrition Recommendations 2023

**DOI:** 10.29219/fnr.v68.10451

**Published:** 2024-02-08

**Authors:** Magnus Domellöf, Agneta Sjöberg

**Affiliations:** 1 Department of Clinical Sciences, Umeå University, Umeå, Sweden; 2 Department of Food and Nutrition and Sport Science, University of Gothenburg, Gothenburg, Sweden

**Keywords:** iron, iron deficiency, anemia, ferritin, nutrition recommendations

## Abstract

Iron absorption from foods is generally lower than that of most other nutrients and is highly variable depending on individual iron status and iron bioavailability in the meal. Several large population groups in the Nordic and Baltic countries are at risk of iron deficiency, including infants, young children, menstruating females, pregnant women as well as vegetarians. Iron deficiency leads to anemia, fatigue, and limited capacity for physical activity. Of particular concern is that iron deficiency anemia in young children is associated with impaired neurodevelopment. A comprehensive literature search has been performed and summarized. New factorial calculations have been performed considering iron losses, iron absorption and iron requirements in various population groups. Recent data on iron intakes and the prevalence of iron deficiency in the Nordic countries are presented. Average requirements and tentative recommended intakes are presented for 12 different population groups. Pregnant women and those with high menstrual blood losses should consume iron-rich food and undergo screening for iron deficiency. Infants should consume iron-rich complementary foods and cow’s milk should be avoided as a drink before 12 months of age and limited to < 500 mL/day in toddlers. Vegetarians should consume a diet including wholegrains, legumes, seeds, and green vegetables together with iron absorption enhancers. There is no evidence that iron intake *per se* increases the risk of cancer or diabetes. Iron absorption from foods is generally lower than that of most other nutrients and can vary between <2 and 50% depending on individual iron status and iron bioavailability in the meal.

## Popular scientific summary

- Iron is essential for many functions in the body, such as forming of hemoglobin that transports oxygen from the lungs to tissues.
- Serum ferritin is the most used single indicator of iron status.
- Iron deficiency is the most common micronutrient deficiency globally.
- Iron deficiency leads to anemia, fatigue and limited capacity for physical activity, and is associated with impaired neurodevelopment in young children.
- Groups in the Nordic and Baltic countries at risk of iron deficiency include infants, young children, menstruating females, pregnant women, and vegetarians.

Iron is essential to virtually all living organisms, its most important biological characteristic being the ability to alternate between two oxidation states – ferrous iron (Fe^2+^) and ferric iron (Fe^3+^) – that can donate or accept one electron, respectively. Iron has many vital functions in the body, the most significant of which is to form the oxygen-binding part of hemoglobin (Hb) that transports oxygen from the lungs to the tissues. Iron is also found in myoglobin, the oxygen-binding protein in muscle fibre. Iron is an important component of many enzymes that transfer oxygen and electrons in a variety of metabolic pathways in all cells of the body, including the brain. For example, iron is necessary for the function of cytochromes that are part of a series of enzymes that couple energy to ATP formation during oxidative phosphorylation.

Iron deficiency (ID) is one of the most common micronutrient deficiencies globally, and is by far the most common cause of nutritional anemia (1), manifested as iron deficiency anemia (IDA). Several large population groups are at risk of ID due to high iron requirements. The relative iron requirements are greatest in infants, young children, adolescents, and pregnant women due to the iron requirements to support growth and blood volume expansion in these groups. Women of fertile age have considerably higher iron requirements than men due to iron losses from menstrual bleeding.

Iron is the most abundant of the trace elements in the human body. An 80 kg adult iron replete body contains about 5 g of iron. Iron has a much lower bioavailability than other nutrients, making dietary factors affecting iron absorption especially important for individuals who have depleted iron stores or ID. However, dietary factors are less important for individuals with high iron stores, in whom iron absorption normally is homeostatically downregulated (2).

## Methods

The review follows the protocol developed within the Nordic nutrition recommendations 2023 (NNR 2023) project (3), Box 1. The sources of evidence used in the review follow the eligibility criteria described previously (4).

Box 1 Background articles for Nordic Nutrition Recommendations 2023

- This article is one of the background articles commissioned as part of the NNR2023 project (3)
- The articles are included in the extended NNR2023 report but, for transparency, these articles are also published in Food & Nutrition Research
- The articles have been peer reviewed by independent experts in the research field according to the standard procedures of the journal
- The articles have also been subjected to public consultations (see report to be published by the NNR2023 project)
- The NNR2023 committee has served as the editorial board
- While these articles are a main fundament, the NNR2023 committee has the sole responsibility for setting DRVs in the NNR2023 project

The systematic review search strategy (see Appendix 1) finally yielded 173 articles, limited of ‘iron’ in title, (Diet OR Dietary OR Food OR Nutrition OR Nutritional) and publication type Systematic review.

A separate literature search was performed in May 2021, which also included randomized, controlled trials. This yielded an initial 535 results with 234 remaining after exclusion. Reasons for exclusion included studies from low/middle income countries, treatment of specific patient groups (not the general population), parenteral nutrition, study not in humans, and publications in other languages than English.

These extensive literature searches did however not cover all topics relevant to iron dietary reference values (DRVs), so we also searched articles relating to physiological iron losses, menstrual iron losses, methods for assessing iron requirements as well as recent reviews and recommendations on iron, most notably the EFSA recommendations from 2015.

## Iron metabolism and bioavailability from diet

### Physiology and metabolism

Iron is the fourth most common element in the earth’s crust but forms highly insoluble iron oxides when exposed to oxygen, making it a challenge for living organisms to utilize iron.

Iron absorption occurs primarily in the duodenum and proximal jejunum, where enterocyte apical-bound enzymes (e.g. cytochrome b reductase) reduce insoluble ferric (Fe^3+^) to absorbable ferrous (Fe^2+^) ions, which are transported into the enterocyte by the transmembrane divalent metal transporter 1 (DMT1). In the enterocyte, iron is either stored as ferritin, some of which is lost when the cells are sloughed, or is transported across the basolateral membrane by ferroportin, after which it is oxidised to Fe^3+^ by hephaestin and then bound by plasma transferrin for transport into the circulation.

In contrast to other sources of iron, heme iron, which is found in meat, poultry, fish, and seafood, has a relatively high and stable bioavailability (about 25%). The molecular mechanisms of heme iron absorption are less well characterized than those for non-heme iron, but once taken up by the enterocyte, it follows the same pathways as non-heme iron.

In order to preserve iron, iron is recycled in the body and humans have no pathway for excretion of surplus iron, a mechanism which is present for other nutrients. This lack of an excretory pathway puts extra demand on strict homeostatic regulation of iron absorption in order to avoid both ID and iron overload.

As ferric iron is poorly soluble and since ferrous iron is a potent pro-oxidant with the capacity to reduce oxygen intermediates to harmful free radicals, all organisms have developed binding molecules (chelators) to transport and store iron and to control its reactivity. Examples are transferrin, which binds iron in plasma, and ferritin, which is the main storage protein for iron and is most abundant in the liver, spleen, and bone marrow.

Iron is also an important nutrient for pathogens such as bacteria and parasites, making iron deprivation an important immune defence strategy for organisms throughout evolution. Indeed, the most important regulator of iron metabolism – hepcidin – was first discovered as a peptide involved in the immune response to bacterial infections. Hepcidin is produced in the liver as a part of the systemic inflammatory response, and binds to the trans-membrane iron transporter ferroportin, thereby blocking iron transport to plasma from enterocytes, macrophages, and hepatocytes (5). This effectively decreases plasma concentrations of iron, depriving the potential pathogen of a growth factor and thereby preventing invasive infection. Since this also deprives erythropoietic cells of iron, a sustained inflammatory state will result in an iron-deficient erythropoiesis and the so-called anemia of inflammation.

However, hepcidin is also the main regulator of iron absorption and iron homeostasis. Since humans have no mechanism for excretion of iron, iron homeostasis is maintained exclusively through regulation of absorption. When iron stores are high, hepcidin synthesis is upregulated, which reduces intestinal iron absorption. Conversely, when iron stores are low or iron requirements are increased (increased erythropoiesis), hepcidin is downregulated, thereby increasing intestinal iron absorption. This mechanism is highly effective, so there is a strong inverse correlation between iron absorption and serum ferritin, which is a marker of iron stores (see below).

### Dietary sources and bioavailability

Meat, poultry, and fish as well as cereals are the main sources of bioavailable iron in a mixed diet. In predominately plant based, vegetarian and vegan diets, beans, lentils, peas, nuts and seeds, and products made from these, wholegrain cereals and dark green vegetables are important iron sources. However, there is large variation between the food sources, with fish having an iron content of 0.2 mg/100 g, poultry 0.4–1.5 mg/100 g and meat 1.0–3.3 mg/100 g (Table 1), refined cereals, wholegrain bread, and wheat 0.7–3.7 mg/100 g, and legumes and products 1.7–3.2 mg/100 g (Table 2).

**Table 1:** Content of iron and heme iron in selection of cooked meat, fish and eggs

Foods	Iron	Heme iron^a^	Heme iron^a^
	mg/100 g	mg/100 g	%
**Meat, poultry, fish and eggs**			
Beef, cooked	2.5–3.3	1.61–2.16	65
Pork, cooked	1.0–1.7	0.38–0.68	39
Chicken, cooked	0.4–1.5	0.11–0.39	26
Chicken nugget, fried	0.5	0.13	26
Salmon, cod, haddock, cooked	0.2	0.04–0.06	26
Egg yolk, cooked	3.1	0.0	0

a According to Balder et al. (6)

**Table 2:** Approximate content of iron and phytate in selected foods

Foods	Iron^a^	Phytate^b^
	mg/100 g	mg/100 g
**Meat replacement products**		
Quorn (mushroom protein)	0.4–0.6	No data
Sausage (soy and wheat protein), Soy mince (soy protein)	1.8–2.5	210–580
Bacon imitation (soy and wheat protein)	20.2^e^	No data
**Bread and cereals**		
Bread whole grain wheat	2.8	430–1,050
Bread white wheat	0.7	30–230
Rice white uncooked	0.1	140–190
Rice brown uncooked	1.3	840–990
Whole grain cereals	2.0–3.7	380–1,460
Bran, wheat	11.0	2,020–5,270
Whole grain flour	3.0	250–1,370
**Nuts**		
Walnuts, hazelnuts, sweet almonds	2.5–3.2	650–3,220
**Seeds**		
Sunflower, Sesame without shell	5.7–7.4	576–1,440
**Legumes**		
Soybean, dry	8.4	1,000–2,220
Soy protein, isolate	10.8^c^	1,400–2,110
Soybean product, tempeh	2.7	670–1,080
Chickpeas, lentils, dry and cooked	1.7–3.2	270–1,260
**Potatoes, vegetables and fruits**		
Potatoes, uncooked	0.4	10–180
Carrots, cabbage, eggplant	0.1–0.3	0^d^
Broccoli	1.1	0^d^
Grean leaves	2.1–3.6	10–70
Berries	0.3–1.1	0^d^
Apples, citrus	0.1–0.2	0–63^d^

^a–d^ Based on different phytate analyses.

a According to the Swedish nutrition data base, https://soknaringsinnehall.livsmedelsverket.se/?soktyp=1#.

b According to Reddy NR in Food Phytates (Reddy, N.R., & Sathe, S.K. (Eds.). (2001). Food Phytates (1st ed.). CRC Press. https://doi.org/10.1201/9781420014419).

c According to Fineli producer food database.

d According to INFOODS (International Network of Food Data Systems) Secretariat. Food and Nutrition Bulletin, vol 25 no. 1 (supplement 2) 2004, https://www.fao.org/infoods/infoods/tables-and-databases/faoinfoods-databases/en/.

e Iron oxide as colorant (E172)

Iron absorption from foods is generally lower than that of most other nutrients, typically around 10–15% from a mixed diet. Iron absorption can vary from < 2% in an iron sufficient individual consuming a meal with high iron content and low bioavailability to about 50% in an iron deficient individual consuming a meal with low iron content and high bioavailability.

Dietary iron consists of heme (from animal tissues) and non-heme iron. Heme iron constitutes about 10–12% of the total iron in a mixed diet, which also has been shown in Nordic populations (7–9). The heme iron intake follows meat consumption and females have lower intake than males (10). The proportion of heme iron of the total iron content in animal-based foods is usually about 50%, but varies between food sources, in cooked foods: 65% in beef, 39% in pork, 26% in chicken and fish (6), see Table 1. Heme iron is generally more efficiently absorbed than non-heme iron and it is generally not affected by other food components. It is also less affected by iron stores than non-heme iron absorption. In a radioisotope study, heme iron absorption was 23% in individuals with average serum ferritin concentrations of 91 µg/L and 35% in individuals with serum ferritin concentrations of 37 µg/L (11). Heme iron absorption is usually estimated to be at least 25%.

Non-heme iron absorption is affected by several dietary components. Single meal radioisotope or stable isotope studies have consistently shown an enhancing effect of ascorbic acid and muscle tissue (meat/poultry/fish) and an inhibitory effect of phytate, polyphenols and calcium.

Ascorbic acid (vitamin C) enhances non-heme absorption by reducing Fe^3+^ to Fe^2+^ at a low pH, in addition to having chelating properties (12). The main effect is achieved by the first 25–100 mg of ascorbic acid in the meal (13). The absorption enhancing effect of ascorbic acid is more pronounced in meals with high concentrations of absorption inhibitors, such as phytate or polyphenols (14–16).

Meals containing lactic acid fermented vegetables have higher iron bioavailability than meals containing fresh vegetables, and the mechanism seems to be an increase in hydrated Fe^3+^ (17).

Muscle tissue from red meat, poultry, fish, and liver enhance iron absorption (18). The nature of this ‘meat factor’ or ‘MPF factor’ (meat/poultry/fish) is uncertain, but a possible explanation is that partially digested peptides, cysteine and histidine residues from muscle proteins, bind non-heme iron and form complexes that are soluble and available for absorption (19). The effect of the ‘meat factor’ is most pronounced in meals containing inhibitors of iron absorption such as phytate.

Some specific probiotic species have recently been suggested to improve iron absorption, even though more studies are needed to confirm this (20).

Phytic acid, also known as phytate, is the main dietary inhibitor of iron absorption. It is found in relatively high concentrations in unprocessed whole-grain cereals, seeds, nuts, and legumes such as soybeans and processed products containing soybean flour or soybean protein (21, 22). The phytate content in some of these foods is shown in Table 2, which is based on different phytate analyses and is presented to give an overview. Like the other dietary inhibitors of iron absorption, phytate binds iron in the gastrointestinal tract, thereby preventing it from being absorbed. Phytate-related inhibition of iron absorption is partly counteracted by ascorbic acid or meat in the meal (14) and soaking or fermentation of cereals and soybeans (23, 24).

Polyphenols are a large group of compounds found in tea, coffee, cocoa, red wine, and some vegetables, legumes, and cereals. Polyphenols from tea seem to have the strongest inhibitory effect on iron absorption.

Calcium has a direct inhibiting effect on non-heme iron absorption and also on heme iron absorption, indicating a possible mucosal rather than luminal effect (25). One glass of milk (165 mg calcium) has been shown to cause a 50% reduction in iron absorption, with a dose-dependent effect up to a consumption of 300 mg calcium in the meal (26, 27). Supplemental calcium has also been shown to reduce iron absorption substantially when taken with meals (28). However, the effect is short-term, and calcium administered 1.5–2 h before the meal does not affect iron absorption (27, 29).

Even though the influence of enhancing and inhibiting factors on iron absorption is very clear in single meal studies, studies of whole diets show varying results. Two-week studies comparing iron absorption from a whole diet containing either enhancing or inhibiting factors of absorption found that iron absorption was twice as high from the diet with the enhancing factors (27). Several studies have shown that the effects of dietary factors on iron absorption are very weak, non-existent, or even reversed in medium-term studies where the intervention lasts for a couple of months (29–34). Recently, Hoppe et al. randomized 55 healthy Swedish females to 12 weeks of high-phytate versus low-phytate bread and could not demonstrate any improvement of iron status in the group consuming low-phytate bread (35). These studies, most of which were performed in iron-replete subjects, demonstrate that the homeostatic mechanisms of iron absorption function extremely well when iron is supplied from a mixed diet and when iron stores are adequate.

However, a diet with high iron bioavailability is likely to be an advantage in individuals with marginal or low ferritin concentrations. In fact, even if iron absorption is up-regulated in this situation, the homeostatic mechanisms may not adjust iron absorption from diet enough to cover iron needs if the iron intake also is low.

### Biomarkers of iron status

#### Ferritin

Serum ferritin (ferritin) is considered to be the best single indicator of iron status and is also the most widely used (36). Ferritin levels are a good indication of the size of the iron stores in the absence of infection and inflammation.

Reference intervals for ferritin are often derived from the 2.5th percentile of the variation in an apparently healthy population. There are a few studies validating ferritin against bone marrow iron stores in adults. One study suggested that a ferritin threshold of < 15 µg/L in healthy women has a sensitivity of 75% and a specificity of 98% of detecting absent bone marrow iron stores, while a threshold of < 30 µg/L had ha higher sensitivity (93%) at the expense of specificity (75%) (37). There is a lack of similar validation studies in children.

Various cut-offs for ferritin have been used in different studies, and recent publications suggest that despite the availability of an international standard for ferritin (38), there is a considerable variation in results depending on the analytical method used (39).

For infants and children, it is important to realize that large changes in iron metabolism occur during the first years of life. Thus, the ferritin cut-off for ID first decreases from 40 µg/L at 0–2 months, to 20 µg/L at 4 months and 10 µg/L at 6–24 months, and then increases to 12 µg/L at 2–5 years of age and 15 µg/L from 6 years to adulthood (1, 36, 40–42).

#### Hemoglobin

In combination with ferritin, this is the most important biomarker of iron status since a reduced concentration of Hb is the definition of anemia.

The reference intervals differ by age and gender: The Hb cut-off for definition of anemia decreases from 135 g/L at 0–1 weeks of age to 90 g/L at 2 months and then increases to 105 g/L at 4–24 months, 110 g/L at 2–5 years and 115 g/L at 6–11 years. In adolescents and adults, gender specific reference intervals are used, the lower cut-offs being 120 g/L for women and 130 g/L for men (see Table 3) (36, 43–45).

**Table 3:** Reference intervals for iron markers (Hb and serum ferritin) in children and adults

Age	0–1 week	2 months	4 months	6–24 months	2–5 years	6–11 years	12–14 years	15 years and above	
Sex								Females	Males
Hb, g/L	135–225	90–140	105–140	105–140	110–140	115–155	120–155	120–155^a^	130–170
Ferritin, µg/L	40–500	40–400	20–300	10–200	12–200	15–200	15–200	15–150	15–200

a Lower limit 105 g/L during 2nd and 3rd trimester of pregnancy.

Pediatric reference values adapted from Ref. (43).

Adult reference values adapted from Ref. (36, 44, 45).

#### Other biomarkers of iron status

Reticulocyte hemoglobin content: This biomarker is useful for detecting ID in adults (46) and children (47–49).

Mean cell volume (MCV), mean cell hemoglobin (MCH), and the red cell distribution width are usually reported from automated blood count devices but are less often used for the diagnosis of ID. MCV is a relatively late indicator of ID and is affected by age, ethnicity, possibly gender, and thalassemia.

Erythrocyte zinc protoporphyrin (ZPP): In a state of ID, iron will sometimes be substituted by zinc in the formation of heme and ZPP will increase. This biomarker is useful for diagnosis of ID and commonly used in fieldwork, but is affected by lead poisoning, malaria, chronic infections, inflammation, and hemoglobinopathies.

Serum iron, total iron binding capacity (TIBC), and transferrin saturation (s-iron/TIBC): These are classical biomarkers of ID, but the main limitation is the considerable diurnal variation of serum iron concentrations due to food intake, requiring a fasting blood sample. TIBC and transferrin saturation are useful for diagnosing iron overload.

Soluble serum transferrin receptor (sTfR) is a useful biomarker of ID and less confounded by inflammation than serum ferritin, although its diagnostic value is still limited in populations where chronic inflammation is common. Also, there is a lack of an international standard and reference intervals vary with different analytical methods.

The sTfR/log(ferritin) ratio is one of the most reliable markers of iron status and is validated in adult men against quantitative phlebotomy and bone marrow staining (the gold standard measurement of iron status). However, the reference values will depend on the analytical method of sTfR.

Hepcidin is a more novel biomarker of iron status. Serum concentrations of hepcidin are usually highly correlated with ferritin but hepcidin may be useful for distinguishing anemia of inflammation and in certain genetic disorders of iron metabolism.

#### The use of biomarkers in practice

The combination of ferritin and hemoglobin is usually recommended for basic screening of IDA (1, 36). For additional sensitivity and specificity, a combination of iron status biomarkers is often recommended, and MCV, transferrin saturation, soluble transferrin receptors, ZPP, reticulocyte hemoglobin and hepcidin are commonly used. However, their sensitivity and specificity for ID are not completely known in different populations and reference intervals for several of these biomarkers differ between laboratories and assays due to the lack of international standards.

A specific limitation of most of these biomarkers is that they are affected by inflammation. In populations with a high incidence of infection and inflammation, for example, sub-Saharan Africa, iron status biomarkers are unreliable in general. Ferritin is especially sensitive for inflammation since it is elevated as an acute phase reactant. However, in the Nordic populations it is a useful marker in general. In individuals with ongoing infection or inflammation (symptoms or elevated C-reactive protein [CRP]), the measurement of ferritin should optimally be postponed until inflammation has subsided. An alternative is to use a higher cut-off for ferritin or to adjust the ferritin value for biomarkers of inflammation (50). For this reason, it is recommended to routinely measure some marker of inflammation, for example CRP, in combination with iron status biomarkers (36).

## Iron deficiency and iron deficiency anemia

If the dietary iron intake is insufficient to meet physiological requirements, iron stores will be mobilised, and ID will develop once the stores are exhausted.

### Prevalence of iron deficiency and iron deficiency anemia

The global anemia prevalence in 2016 was 42% in children < 5 years, 40% in pregnant women and 33% in non-pregnant women (30). Iron deficiency is the single most common cause of anemia and is estimated to cause between 25 and 50% of global anemia cases (31, 32). However, the prevalence of ID and IDA varies greatly by geographic region and incidences in the Nordic countries are considerably lower than the global average. Nevertheless, some population groups, especially women and young children, are at high risk of ID also in the Nordic countries. It is notable that the exact prevalences of ID and IDA depend on the cut-offs chosen for ferritin and hemoglobin, see next section.

#### Young children

Due to high growth-related iron requirements, young children < 5 years are at high risk of ID, especially around the age of 12–36 months. A study from 2019 showed that the prevalence of ID in 18-month-old Danish children was 15% (defined as ferritin < 12 mg/L) (33). This is similar to previous studies from Norway, Sweden and Iceland from 2004 to 2011, which showed an ID prevalence of 6–18% in 12-month-olds (51–53) and 13% in 2-year-olds (51). This is also similar to a study of children aged 12–36 months from Germany, the Netherlands and United Kingdom, which found that the prevalence of ID was 12% (54). The prevalence of IDA was only reported in a few of these studies, but in the Icelandic study from 2011 (53), no case of anemia was found at 12 months, while in the Norwegian study from 2004, the prevalence of IDA at 12–24 months was 5% (51). Iron status in young children in the Nordic countries seems to have improved since the 1990s, probably due to improved dietary advice, including the advice not to use cow’s milk as a drink before 12 months of age (53).

#### Adolescents

Adolescents are at increased risk of ID, especially adolescent girls due to the onset of menstrual blood loss. A few recent studies presenting prevalence of ID and IDA among adolescents in the Nordic region were found. The results vary due to age groups, choice of cut-off values and how confounding of results due to previous infection was handled.

A Norwegian study included 15–17-year old girls and boys at baseline with a 2-year follow-up of prevalence of ID and IDA (55). ID was defined as mild, moderate and severe based on ferritin cut-offs ≥12 to <16 µg/L, ≥ 5 to < 12 µg/L and < 5 µg/L, respectively. The prevalence of ID in the baseline study among girls was 9.6, 14.5 and 3.6%, and among boys 1.6, 3.5 and 1.6%, respectively. Two years later at age 17–19 years, the corresponding figures for girls were 7.2, 11.7 and 0.7%, and for boys 0.8, 0.8 and 0%. Defining IDA in girls with Hb < 12 g/dL and SF < 12 µg/L, and in boys with Hb < 13 g/dL and SF < 12 µg/L gave a prevalence of IDA of 9.6% among girls and 0.6% among boys (55).

In a Swedish national study from 2016 to 2017 a subsample was tested for ID defined by ferritin < 15 µg/L and excluding subjects with CRP ≥ 5 mg/L (56). The study included children/adolescents from 5th grade (11–12 years), 8th grade (14–15 years), and from the second year in high school (17–18 years). The prevalence of ID in girls was 10, 30 and 26% in respective age group. The corresponding ID prevalence for boys was 3, 11 and 2% in the respective group.

#### Adults

Among women of childbearing age, the prevalence of ID (ferritin < 15 µg/L) was 29% in a subsample of a Swedish national study (9). In women in early pregnancy, anemia was found in 5.9% and ID (ferritin <15 µg/L) in 33% in a Norwegian study, in which also large differences in prevalence were shown according to ethnic origin (57). Only a few studies have been conducted on iron status in elderly populations in the Nordic countries, and there is a lack of recent studies. The prevalence of low Hb among the elderly (>65 years) has been found to be 0–5%. ID is relatively uncommon in 70-year-olds, and even more uncommon in healthy 80- and 85-year-olds, but high iron stores (ferritin >300 µg/L) have been observed in 8.7% of elderly men and 3.7% of elderly women (58, 59).

### Risk factors for ID and IDA

Dietary risk factors for ID and IDA in early childhood include prolonged exclusive breastfeeding (> 6 months), a high intake of cow’s milk and a lack of iron-rich diet (meat, iron fortified products) at 6–36 months of age. Non-dietary risk factors for ID and IDA in early childhood include low birth weight (< 2,500 g), early umbilical cord clamping, male sex, and low socioeconomic status.

A low dietary iron intake is a risk factor for ID in all age groups. Individuals on a vegetarian or vegan diet are a special risk group, see next.

Risk factors for ID and IDA in fertile women include large menstrual blood losses, and increased iron requirements during pregnancy.

Gastrointestinal blood loss is the most common cause of ID in men and postmenopausal women.

Elite athletes, especially in endurance sports, have increased risk of ID, which is probably multifactorial due to low-grade inflammation, increased iron losses and increased iron requirements for erythropoiesis (60).

### Effects of iron deficiency

ID is most commonly asymptomatic, even though some studies suggest that ID may be associated with reduced attention and concentration (61), or lowered endurance during cognitive-demanding tasks (62). In a meta-analysis of four studies including iron deficient but non-anemic adults, iron supplementation resulted in reduced subjective fatigue scores, however, no effect on objective measures of physical capacity were found (63). ID is also associated with restless legs syndrome (64).

IDA can also be asymptomatic if mild, but pallor, fatigue, limited capacity for physical activity, palpitations, dizziness, and headache are typical symptoms, which increase with the severity of anemia.

Of particular concern is the observation that children with IDA have impaired neurodevelopment (65, 66). Intervention studies have yielded conflicting results, but recent meta-analyses show positive effects of iron supplements (67, 68). However, iron supplementation seems to be more effective for reversal of symptoms in older children, suggesting that IDA in infants and young children may result in irreversible impairment of neurodevelopment (65). Thus, prevention of IDA in young infants should be a high priority.

### Stages of iron deficiency

When an inadequate amount of dietary iron is absorbed, due to low intake and/or low bioavailability relative to requirements, the development of ID proceeds continuously from normal iron status to IDA (5). Initially body iron stores diminish, which is reflected in a decreasing concentration of ferritin. When iron stores start to become depleted, ID in tissues develops and this leads to increasing levels of transferrin, reduced transferrin saturation and increased sTfR. Iron deficient erythropoiesis results in increased ZPP, and lower reticulocyte hemoglobin content. Next, MCV is reduced and finally the Hb level starts to decrease, and if the negative iron balance is not corrected anemia develops. However, in the clinical setting of ID, individual changes in iron status biomarkers do not necessarily follow this strict order. Thus, a combination of iron status biomarkers is more useful than a single biomarker.

## Health risks of a high dietary iron intake

### Iron and risk of diabetes

The scoping review of systematic reviews and meta-analyses undertaken for NNR 2023 suggested that a high heme iron intake and high ferritin levels are associated with higher risk for type 2 diabetes (T2D). The relative risk (RR) elevation of heme iron intake on T2D for the lowest and the highest quintile of heme iron intake was RR 1.20–1.33 (69–72). Bao et al. (69) reported median heme iron intakes 2.39 mg/day and 0.56 mg/day in the highest versus the lowest level of heme iron intake. No effect or an inverse effect on the risk of T2D was seen for non-heme iron and for total iron intake (69, 71). Three systematic reviews and meta-analyses evaluating effects of iron stores on T2D showed risk estimates RR: 1.63–1.73 for the highest ferritin levels versus the lowest (69, 70, 73). An association between high ferritin concentrations and T2D has been shown in recent meta-analyses, OR 1.43 for high ferritin concentration, while it was 1.20 for a median ferritin concentration, compared to the lowest level (74).

The relationship between iron intake and body iron stores in prospective studies with risk for developing gestational diabetes mellitus (GDM) is presented in systematic reviews and meta-analyses. No effect was found for total dietary iron intake, RR 1.01 (75), and non-heme iron intake (76, 77). In contrast, dietary intake of heme iron was significantly associated with GDM risk, RR 1.53–1.65 (75, 76). Also, ferritin was significantly related to GDM risk, RR 1.41–3.22 (75, 76, 78). A meta-analysis with less standardized inclusion criteria reported RR 1.58 for ferritin and 1.48 for heme iron intake in relation to risk for GDM (79).

Thus, meta-analyses of observational studies present probable evidence for the association of heme iron intake and ferritin levels with the risk of T2D and GDM. The observed association between ferritin concentrations and risk of T2D can be influenced by a number of confounders including age, sex, and ethnicity (74). Furthermore, ferritin can be increased due to for example inflammation, obesity, and/or liver disease and ferritin may be elevated with a normal CRP. Importantly, heme iron is directly related to the intake of red meat and processed meat as well as with other lifestyle factors, so these associations might not be causal.

Because there is no evidence that total dietary iron intake is associated with increased risk of T2D or GDM, this does not have any implication for recommended daily intakes of iron.

Recent studies show conflicting results regarding iron intake and the risk of developing T1D in children; One study suggested that high iron intake was associated with an increased risk of islet autoimmunity in children with high genetic risk of T1D (80). Another study showed that higher iron intake at the time of islet autoimmunity was associated with a lower risk of progression to T1D (81). A recent review found no conclusive evidence for an association between iron intake and type 1 diabetes (T1D) (82).

With regard to the association between heme iron intake and the risk of diabetes, this relates to meat intake, and we refer to the section on red meat and processed meat in NNR 2023.

We conclude that there is currently no convincing evidence that dietary iron intake is a causal factor that would explain increased risk of T2D, GDM or T1D, but that this area needs more research.

### Iron and risk of cancer

Due to the known pro-oxidant effects of iron, the known association between hemochromatosis and liver cancer, and observed associations between meat intake and cancer, iron has been suggested as a risk factor for different types of cancers. Possible associations between iron intake, iron status and the risk of various cancers have been investigated in many studies (83–86).

Some meta-analyses have shown significant associations between red meat intake and breast cancer, endometrial cancer, colorectal cancer, lung cancer as well as hepatocellular carcinoma. Furthermore, even stronger associations have been shown between intakes of processed meat and the risk of colorectal cancer, lung cancer and breast cancer (87, 88). Other meta-analyses have shown only very weak associations between red and processed meat consumption and cancer outcomes (89). There is a lack of evidence from intervention trials that show any effect of lower red meat intake on cancer outcomes (90). The World Cancer Research Fund (WCRF) in 2018 concludes that there is limited evidence that consumption of red meat and processed meat increases the risk of colorectal cancer (RR 1.10, 95% CI 1.02–1.18, and 1.13, 95% CI 1.00–1.29, in males and females, respectively. They also concluded limited evidence for foods containing heme iron and risk for colorectal cancer (RR 1.22, 95% CI 1.12–1.30) (91). On the contrary, the global burden disease study reported low effect of a diet high in red or processed meat (92). Most authorities, including the Nordic nutrition recommendation (NNR), recommend a limitation of the consumption of red meat and processed meat, see section on red meat in NNR 2023. However, it is much less clear if iron intake has any causal effect in relation to this. One possible mechanism for the association between meat intake and cancer are the mutagenic compounds formed when cooking meats at high temperatures (91) but it has also been suggested that heme iron intake can have DNA damaging effects (93). A systematic review of the mechanistic studies of the link between heme iron intake and risk of colorectal cancer showed that these studies were based on levels of heme iron that were much higher than those in normal human diets, and concluded that there is insufficient evidence to confirm a mechanistic link (94). A meta-analysis of 59 studies (95) showed a borderline significant association between iron intake and colorectal cancer (RR 1.08, 95% CI 1.00–1.17) and no significant association with breast cancer or lung cancer, the latter which was supported by another recent meta-analysis (96). A study based on 1,126 cases and 1,173 matched controls did not show any significant association between serum ferritin concentrations and colorectal cancer (97). A study on esophageal cancer showed that there was a positive dose-response relationship with heme iron intake but a negative dose-response relationship with total iron intake (98). A study on breast cancer showed that heme iron intake was significantly associated with increased risk, whereas no associations were found for dietary iron, supplemental iron, or total iron intake (85). Even though the risk of hepatocellular carcinoma is known to be increased in individuals homozygous for hemochromatosis (99), the relation between primary liver cancer and iron intake in the general population is less clear. A recent meta-analysis showed an association between high serum ferritin levels and primary liver cancer but the associations were subject to heterogeneity and it was concluded that further confirmatory studies are needed (100).

WCRF does not not recommend to completely avoid meat even if significant modest risk increases for colorectal cancers have been observed, because meat can be a valuable source of nutrients, in particular protein, iron, zinc and vitamin B_12_ (91, 101).

We conclude that there is no convincing evidence that dietary iron intake is associated with increased risk of colon cancer, lung cancer, breast cancer, esophageal cancer, or other cancers, and that there is no evidence to support an UL of iron intake based on cancer risk.

## Estimation of iron requirements

Recommendations for iron intake cannot be based directly on health outcomes, since there is a lack of well-powered, randomized, controlled intervention trials with a sufficiency long term follow-up. In addition, observational studies of associations between iron intakes and health outcomes cannot form a direct base for recommendations due to uncertainties in intake measurements, poor correlation between intake and iron status, and the presence of confounders that prevent the determination of dose-response relationships and the assessment of risks associated with deficiency or excess.

Thus, the NNR are based mostly on factorial calculations, considering the following factors: 1) iron losses, 2) iron absorption and 3) iron requirements for growth (in children and pregnant women).

### Iron losses and needs for growth

#### Basal iron losses

In non-menstruating, non-pregnant healthy adults, small amounts of iron are lost via skin, hair, sweat, urine, and feces. This is usually called basal iron losses. Larger individuals have higher iron losses, not due to higher fat mass but due to higher body surface area and thus larger losses of exfoliated skin cells.

There are two commonly cited studies that have estimated basal iron losses in different populations of healthy adults, both using radioisotope dilution measurements: Green 1968 and Hunt 2009, which yield very similar results, showing that the average iron losses are close to 1 mg/day or 12–14 µg/kg/day (102, 103).

We estimated average basal iron losses in adults to 14 µg/kg/day, and the upper limit of variation was set using 15% CV. The alternative of using 12 µg/kg/day for adult men and the upper limit of variation, from Hunt 2009 (excluding a single case with the highest iron losses), yields very similar results for adult men. We consider that the Hunt reference cannot be used to determine losses for menstruating women (due to menstrual blood losses) and postmenopausal women (very small sample size, *n* = 5) (103).

In infants and young children up to 5 years of age, iron losses have been estimated to be higher than in adults per kg body weight: 22 µg/kg (104). In children older than 5 years and adolescents, iron losses are estimated to be 12 µg/kg/day (105).

#### Menstrual blood losses

Women of reproductive age have high iron losses due to menstruations, which is the main cause of the high and variable iron requirements in this population group.

The normal variation of menstrual blood loss in healthy Swedish women was described in a series of articles by Hallberg et al. in the 1960s (106,107,108). The largest of these studies (108), which included 476 women, showed that the average blood loss per menstrual cycle was 43.4 mL, the median was 30 mL and the 90th percentile was 84 mL. This translates to 0.48 mg and 1.35 mg iron per day, based on a 28-day cycle. The 95th percentile was 1.90 mg. Blood losses were higher in older women, around 50 years of age, approaching the menopausal transition. Excessive menstrual blood loss is a well-known risk factor for ID anemia, and Hallberg suggested that losses > 80 mL/cycle should be defined as pathological.

While contraceptives are commonly used today, they were not widely used in the 1960s. Oral contraceptive use is associated with lower menstrual blood losses (109). Harvey et al. presented data in 2005 on 90 women aged 18–45 years, of which 35.5% used oral contraceptives and 5.5% had an intrauterine device. In that study, the mean (SD) iron loss was 0.43 (0.45) mg/day and the 50th, 90th, 95th and 97.5th percentiles were 0.26, 0.91, 1.32 and 1.71 mg/day, translating to per-cycle blood losses of 16, 57, 82 and 106 mL, respectively (110).

Hunt performed a detailed study on a very small sample of premenopausal women (*n* = 13) (103). In that study, menstrual blood losses varied between 1 and 124 mL per cycle, translating to iron losses between 0.015 and 1.86 mg/day, with a geometric mean (SD) of 0.28 (3.7325). This geometric distribution translates to a 97.5th percentile of 3.73 mg/day, which probably only reflects the very small sample size in this study.

In summary, based on the largest datasets, average iron losses due to menstrual bleedings seem to be about 0.45 mg/day (Hallberg median 0.48 mg, Harvey mean 0.43 mg/day). The upper limit is more difficult to define but the 90th percentile according to Hallberg was 1.35 mg/day and the 95th percentile according to Harvey was 1.32 mg/day (individual data from the Harvey study is presented in Appendix A in EFSA 2015). Considering that oral contraceptives were not commonly used in the 1960s, the latter number may best reflect the current situation in the Nordic countries. For calculations, we have used an average Hb concentration of 135 g/L and an Hb iron content of 3.34 mg/g, resulting in a conversion factor of 0.45 mg Fe/mL blood.

Similar to EFSA 2015, we consider the 95th percentile, rather than the 97.5th, to be the adequate target for premenopausal women. For the 5% of women with the highest menstrual blood losses, > 80 mL/cycle, which can be considered to be hypermenorrhea, we recommend screening (Hb, ferritin) and iron supplements when indicated. A special risk group are women around the age of 50 years who are undergoing transition to menopause.

During lactation, the quantity of iron secreted in breast milk is approximately 0.24 mg/day, based on an iron concentration in mature milk of 0.3 mg/L (111) and assuming an average milk volume of 0.8 L/day (112). However, since lactation delays the onset of menstruation, the increased iron losses through breast milk is more than covered by the absence of menstrual blood loss. Thus, no separate recommendation for iron requirement is given for lactating women.

#### Iron needs for growth

Since iron is required for tissue growth and expanded blood volume, young children, adolescents, and pregnant women have increased iron needs.

An exception is the first 6 months of life: When the newborn emerges from the low-oxygen intrauterine environment out into the oxygen-rich atmosphere, erythropoiesis is halted and the blood hemoglobin level falls from an average of 170 g/L to about 120 g/L during the first 6 weeks of life. During this period, iron is transferred from hemoglobin to iron stores, making the infant self-sufficient with regard to iron until the infant has doubled his or her birth weight, which occurs at about 6 months of age in a term, normal-birth-weight infant. Thus, exclusive breast-feeding during this period will meet iron requirements despite the low concentration of iron in breast milk (0.3 mg/L). However, infants with low birth weight will deplete their iron stores more rapidly and need iron supplements during the first months of life, see next.

Between 6 and 12 months of age, continued rapid growth and initially depleted iron stores lead to higher iron requirements per kilogram body weight than during any other period of life (42). Based on a blood volume of 80 mL/kg, tissue iron of 7 mg/kg, and a minimal amount of iron stores of 2 mg/kg at 12 months, an average body weight of 7.9 kg at 6 months and 10.1 kg at 12 months, the requirement of absorbed iron for growth is 0.54 mg/day, resulting in an average total dietary iron requirement of 0.74 mg/kg/day (Table 4).

**Table 4:** Iron requirements based on factorial calculations and recommended intakes of iron.

	Weight^c^ (kg)	Weight gain (kg/year)	Fe required for growth^d^ (mg/day)	Basal iron losses^e^ (mg/day)	Menstruation losses of iron (mg/day)	95th percentile	Total requirements of absorbed Fe (mg/day)	Upper limit of variation^f^	Bio-availability (%)	Average iron requirement (mg/day)	Tentative RI (mg/day)	Rounded RI (mg/day)	EFSA 2015 PRI	NNR 2012 RI	IOM 2001
6–12 months^a^	9.0	4.4	0.54	0.20			0.74	0.96	10	7.4	9.6	10	11		11
1–3 years^b^	13.6	2.3	0.26	0.30			0.55	0.72	10	5.5	7.2	7	7	8^aa^	7
4–6 years^b^	20.7	2.4	0.26	0.25			0.51	0.66	10	5.1	6.6	7	7	8^aa, bb^	10^cc^
7–10 years^b^	30.8	3.3	0.36	0.37			0.73	0.94	10	7.3	9.4	9	11	9^bb^	
11–14 year girls	46.5	4.4	0.46	0.56	0.45	0.89	1.47	1.90	15	9.8	12.7	13	13^dd^	11	8^ee^
11–14 year boys	48.2	5.6	0.74	0.58			1.32	1.71	15	8.8	11.4	11	11^dd^	11	8^ee^
15–17 year girls	57.9	1.7	0.18	0.69	0.45	1.32	1.32	2.19	15	8.8	14.6	15	13^dd^	15	15
15–17 year boys	65.6	4.1	0.54	0.79			1.33	1.72	15	8.8	11.49	11	11^dd^	11	11
Adult men	73.4			1.03			1.03	1.34	15	6.9	8.9	9	11	9	8
Women of reproductive age	64.2			0.90	0.45	1.32	1.35	2.22	15	9.0	14.8	15	16	15	18
Postmenopausal women	62.5			0.88			0.88	1.14	15	5.8	7.6	8	11	9	8
Pregnant women	76.4		1.91	1.07			2.98	3.87	15	19.9	25.8	(26)^ff^	16	Suppl	27

a 6.0–11.9 months.

b 1.0–3.9 years, 4.0–6.9 years, 7.0–10.9 years etc.

c Calculation weight (average weight during period).

d From 1 year based on total body iron content 40 mg/kg up to 11 y (pre-puberty), 48 mg/kg in adolescent boys and 38 mg/kg in adolescent girls [EFSA].

e Basal iron losses 22 µg/kg/day up to 3 years, thereafter 12 µg/kg/day. For women, 14 µg/kg/day has been used.

f We used 97.5th percentiles except for adolescent girls and women of reproductive age for which values are 95th percentiles. A CV of 15% was used in the absence of data.

aa 2–5 years

bb 6–9 years

cc 4–8 years

dd 12–17 years

ee 9–13 years

ff Screening and supplementation is recommended.

After 1 year of life, growth is slower and almost linear until puberty. The iron requirements for growth have been calculated with the factorial approach, using the NNR2023 weights for the intervals 1–4 years etc. since the 1–3-year range includes the period up to the 4th birthday. Total body iron was estimated to 40 mg/kg at 1–11 years (pre-puberty) (105). The average iron requirement for growth is estimated to be 0.26–0.36 mg/day during this period (Table 4).

During puberty, growth is accelerating so iron requirements for growth increase during adolescence (11–18 years). Total body iron was estimated to 48 mg/kg in adolescent boys and 38 in adolescent girls (105). The average iron requirement for growth during the whole period of 11–18 years is estimated to be on average 0.64 mg/day for boys and 0.32 mg/day for girls (Table 4). However, as described here, blood loss from menstruation is the main driver of iron requirements in adolescent girls.

During the 280 days of a normal pregnancy, 270 mg of iron is required for fetal growth, 90 mg for the placenta and umbilical cord and 175 mg for the average blood loss at delivery (113, 114). This leads to an additional need of absorbed iron of 1.91 mg per day during pregnancy, in addition to basal iron losses.

### Iron absorption

Iron absorption from the diet is a key factor determining dietary iron requirements, and it is to a large extent homeostatically regulated by the size of the body iron stores; it is well known that iron absorption is inversely proportional to ferritin concentration (115). There is a several-fold difference in iron absorption from a meal between an individual who is iron deficient and another individual with large iron stores. In addition, dietary iron bioavailability is dependent on dietary factors, that is the proportion of heme iron and non-heme iron in the diet and the presence of enhancers and inhibitors of non-heme iron absorption. These dietary factors are probably most important in individuals with low iron stores. In a systematic review of 19 non-heme iron absorption studies from whole diets performed in Europe, USA, and Mexico, most diets had low bioavailability of non-heme iron with a typical absorption of 5–8% (116). The lowest absorption was 0.7%, observed in men with mean ferritin 100 µg/L consuming a low-bioavailability diet. The highest absorption of 22.9% was observed in women with ID (mean ferritin 6.4 µg/L) consuming low bioavailability diet with added vitamin C. Heme iron is well absorbed (typically 25%, variation 10–40%) (117) and absorption is less influenced by dietary enhancers and inhibitors.

Several different algorithms have been developed for prediction of non-heme iron absorption. The original Swedish algorithm (118) was based on iron absorption data from single meals labelled with radioiron, adjusted to the absorption of a reference dose. This complex formula, which considered estimated effects of dietary enhancers and inhibitors of iron absorption for each meal, gave similar results as the measured absorption. Other algorithms have been developed using absorption data from single meals (119, 120). More recently, there have been attempts to develop algorithms based on complete diets. Armah et al. used data on 53 individuals (19–38 years) from 4 diet studies (121). Iron absorption was measured using extrinsic radiolabelling during three 1-week periods with different diets, giving a total of 159 measurements. Ferritin was by far the strongest predictor of iron absorption with a partial R^2^ of 0.35. A formula was constructed based on the multiple linear regression model to quantify the effect of different factors on non-heme iron absorption:

*Ln non-heme iron absorption* (%) = 6.294 – 0.709 *ln* (SF) + 0.119 *ln* (C) + 0.006 *ln* (MFP + 0.1) – 0.055 *ln* (T + 0.01) – 0.247 *ln* (P) – 0.137 *ln* (Ca) – 0.083 *ln* (NH)

where SF is serum ferritin (μg/L), C is ascorbic acid (mg), MFP is meat, fish and poultry (g), T is tea (number of cups), P is phytate (mg), Ca is calcium (mg) and NH is non-heme iron (mg).

Pooled data from 40 individuals undertaking iron absorption studies of identical design showed average non-heme absorption from a self-selected diet, a low bioavailability diet (high calcium, low vitamin C, no meat), and a high bioavailability diet (low calcium, high vitamin C, high meat) of 7.09, 7.17and 9.92%, respectively (116).

The current US iron recommendations for iron intake (122), which have not been updated since 2001, were based on a non-heme iron absorption of 7.4% from self-selected diets in a population with mean serum ferritin of 34 µg/L. Adjusting to a ferritin level of 15 µg/L resulted in a bioavailability of non-heme iron of 16.8%. Adding heme iron intake (10% of total iron intake, 25% absorption), the estimated overall iron bioavailability in the mixed American or Canadian diet was calculated to be 17.6% for non-pregnant adults and most children. In infants, however, a diet with little meat and rich in in cereals was estimated to have a lower bioavailability of 10%.

EFSA (2015) used an alternative method to calculate total dietary iron bioavailability, using data on dietary iron intakes and ferritin from a UK study population of 495 men and 378 premenopausal women (123). Iron absorption was not measured but calculated using a probability model based on estimated absorbed iron requirements (124) together with ferritin levels and total iron intakes in the study population. Neither the intake of heme and non-heme iron, nor dietary enhancers and inhibitors were included in the model. The EFSA method resulted in significantly higher estimated iron absorption at comparable ferritin levels, compared to estimates by other authorities: At ferritin 15 µg/L, the iron absorption in women was estimated to be 31% and the iron absorption in men could not be estimated at that ferritin level due to too few men having low ferritin levels (123). Thus, the EFSA instead normalized to the ferritin level of 30 µg/L, which resulted in an estimated total dietary iron absorption of 18% in women and 16% in men.

#### NNR2023 conclusion on iron absorption

In Nordic population studies, the following typical daily intakes are found among adults: vitamin C (93–113 mg/day), meat (red meat 68–172 g/day), fish and poultry (fish and seafood 27–79 g/day, poultry 20–43 g/day), tea (40–238 g/day), calcium (811–1,188 mg/day), total iron (9.4–13 mg/day) and non-heme iron (8.3–11.7 mg/day) (10). There is a lack of Nordic population-based studies regarding phytate intake, so a typical intake similar to that of US omnivorous adult males and females (range 585–781 mg/day) (119) is assumed.

Unfortunately, there is also a lack of large, population-based studies in Nordic adults showing both iron intakes and ferritin concentrations. We have used two datasets: 1) The above-mentioned UK population-based study of 873 adult men and premenopausal women (123), with an average ferritin of 89 µg/L and iron intake of 11.9 mg/day and 2) The Swedish population-based study of 711 adolescents aged 14–17 years (56) with an average ferritin of 38 µg/L and iron intake of 8.6 mg/day.

Applying the Armah formula on the above numbers (midpoints of ranges and assumed 90% non-heme iron) yields an average non-heme iron absorption of 2.5% based on the UK data and 4.8% based on the Swedish adolescent data. After normalizing to a ferritin concentration of 15 µg/L, using the well-established ratio method (125) and the conservative estimates that 10% of the intake is heme iron (124) with a bioavailability of 25% (126), the resulting total iron absorption from the diet was calculated to be 16.1% based on the UK population (123) and 13.4% based on Swedish adolescents (56). The average of those two estimations is 14.8%.

Thus, we do not see a reason to change the assumption of 15% iron absorption from the NNR2012. Using the slightly higher EFSA assumption of 16% absorption in men and 18% in women, would result in correspondingly lower recommendations, which might not be sufficient for individuals at risk. Furthermore, there is no evidence that men and women have different iron absorption at the same ferritin level.

It should be noticed that a large majority of the population will have a much lower actual iron absorption from a mixed diet (around 5–7%) and that iron absorption is upregulated toward 15% only in individuals at risk of ID, that is those whose ferritin concentrations have decreased toward 15 µg/L. In iron deplete subjects, absorption will increase further.

There is very limited information on iron absorption in children and like the EFSA we have used a conservative estimate of 10% absorption for children up to 10 years (105). Iron absorption in adolescents have been assumed to be similar to that in adults, even though the absorption might be slightly lower as shown in the above example.

### Calculated iron requirements

Calculated iron requirements in different population categories are presented in Table 4. Upper ranges (tentative RI) are based on the 97.5th percentile of the variation in requirements, when not otherwise specified. A CV of 15% has been used in the absence of data. There are no studies of the overall variation in iron requirements depending on the combination of variation in basal losses, menstrual blood losses, growth, and iron absorption, but these factors have only been studied separately. Also, the variation in iron absorption counteracts the other variations due to homeostatic regulation. We have based the estimated population variation on the single factor (basal losses, menstrual blood losses or growth), which represents the largest variation in the respective population category. Some population categories are described more in detail next.

#### Adult women of reproductive age

In addition to the basal iron losses, women of reproductive age also have menstrual blood losses, the latter representing most of the variation in iron losses. As discussed here, the average absorbed iron requirements due to menstrual blood losses are estimated to be 0.45 mg/day and the 95th percentile is 1.32 mg/day. Similar to EFSA 2015, we consider the 95th percentile, rather than the 97.5th percentile, of iron losses to be the adequate target for women of reproductive age. For the 5% of women with the highest menstrual blood losses, > 80 mL/cycle, which can be considered to be hypermenorrhea, a reasonable approach could be screening (Hb, ferritin) and iron supplements when indicated. Screening and treatment of women with high menstrual blood losses has been shown to improve quality of life (127). In addition to adolescent girls (see next), a special risk group are women around the age of 50 years who are undergoing transition to menopause.

Based on menstrual blood losses, basal losses of 0.90 mg/day and 15% iron absorption, the calculated average iron requirement is 9.0 mg/day and the 95th percentile is 14.8 mg/day.

In a Swedish national study from 2010 to 2011, 449 females of childbearing age had an average total iron intake of 9.3 mg/day and 29% had ID (ferritin < 15 µg/L) (9). This suggests that a significant proportion of the population do not achieve the RI. However, there is a lack of recent data from the Nordic countries, so such data is needed.

#### Infants 0–6 months

Traditionally, no recommendations for iron intake are given for this age range since the healthy, normal birth weight infant has very limited requirements of dietary iron during this period and since breastfeeding is the primary recommendation. Infant formulas should be iron supplemented, even though the optimal amount of iron supplementation is still controversial (128). Delayed cord clamping is recommended.

For infants with low birth weight (< 2,500 g), iron supplements are recommended, at a dose of 1–3 mg/kg/day depending on the birth weight (129).

#### Infants and toddlers

For 6–12 months, based on an estimated average requirement of absorbed iron of 0.74 mg/day and 10% bioavailability, the calculated average iron requirement is 7.4 mg/day. Assuming a CV of 15 %, this results in a tentative RI of 9.6 mg/day. The NNR2012 did not specify iron recommendations for this age range. The EFSA gives a slightly higher RI of 11 mg/day based on a higher assumed accumulation of iron stores.

For 1–3 years, similar factorial calculations result in a tentative RI of 7 mg/day, which is slightly lower than the NNR2012 (8 mg/day) but equal to the EFSA recommendation.

Even though the prevalence of IDA is low in Nordic infants and toddlers, the prevalence of ID is still relatively high. A recent Danish study (33) found an ID prevalence (ferritin < 12 µ/L) of 15% in 370 18-month-olds. A recent study in Finland (130) in 766 infants and toddlers, showed a prevalence of ID (ferritin < 10 µg/L) of 14% at 12 months and 20% at 24 months of age. Average iron intakes in that study, estimated from FFQs at 12 months were 6.3 mg/day, suggesting that most infants did not achieve the RI.

International recommendations emphasise dietary advice for prevention of ID in infants and toddlers at 6–24 months of age: 1) Iron-rich complementary foods starting at 6 months; 2) Avoid cow’s milk as a drink before 12 months of age and 3. Limit the intake of cow’s milk to < 500 mL/day in toddlers (42). Iron-rich complementary foods for this age group include iron-fortified milk products (follow-on formulas) and iron-fortified cereal products for example gruel and cereal-based drinks (‘välling’).

#### Adolescent girls

The main driver of the higher iron requirements in adolescent girls is the onset of menarche, which occurs on average at 12.7 years of age. It is thus a challenge to define iron requirements in the age group 11–14 years (covering the period up to the 15th birthday).

Based on the factorial calculations described here and in Table 4, the calculated average iron requirements in adolescent girls were estimated to be 9.8 mg/day at 11–14 years and 8.8 mg/day at 15–17 years. The upper limit of variation, as defined by the 95th percentile results in a RI of 15 mg/day at 14–17 years. Since about half of the girls in the age range 11–13 years have not yet reached menarche, the variation was halved in this age group, resulting in an overall RI of 13 mg/day. However, for girls who are known to have reached menarche in this age range, the higher RI of 15 mg/day can be applied.

The tentative RI for 11–14 years of 13 mg/day is higher than the NNR 2012 (11 mg/day) but the same as EFSA 2015. The suggested RI for 15–17 years of 15 mg/day is the same as NNR 2012 but higher than EFSA 2015 (13 mg/day).

Iron deficiency and ID anemia are common in this population group. In a recent Norwegian study of 408 adolescent girls, the prevalence of ID (ferritin < 16 µg/L) was 28% and the prevalence of IDA (Hb < 120 g/L and ferritin < 12 µg/L) was 10%. In the Swedish study ‘Riksmaten Ungdom’ (56), the total iron intake was 8 mg/day in adolescent girls and the proportion of ID was 26–30%. This suggests that the majority of adolescent girls have iron intakes below the RI.

Similar as for adult women, adolescent girls with the highest menstrual blood losses are at high risk of ID and we recommend screening (Hb, ferritin) and iron supplements when indicated.

#### Postmenopausal women

The average age of menopause is 51 years. Thus, 50% of women are still menstruating at 51 years but none at 70 years, making it difficult to give a recommendation for the age range of 51–70 years. For women still menstruating, the recommendation for women of reproductive age (25–50 years) should be used.

#### Pregnant women

In addition to basal iron losses, which amount to approximately 300 mg during the 280 days of a normal pregnancy, 270 mg of iron is required for the fetus, 90 mg for the placenta and umbilical cord and 175 mg for the average blood loss at delivery (113, 114). This leads to an additional need of absorbed iron of 1.91 mg per day during pregnancy, giving a total need of 2.98 mg/day. Assuming 15% absorption, the calculated average iron requirement is 19.9 mg/day. Assuming 15% CV of the pregnancy-related requirements, the 97.5th percentile is 26 mg/day. This is similar to the reasoning in NNR2012 (even though a precise RI was not reported) and similar to the US recommendations (122) but differs considerably from the EFSA 2015 PRI, which is 16 mg/day.

IDA is common during pregnancy (57) and since it is very difficult to achieve dietary iron intakes of more than 20 mg/day, in addition to dietary advice, it is usually recommended that pregnant women are screened for IDA (Hb, ferritin) and that iron supplements are given when indicated (45).

This recommendation to screen and treat is consistent with the most current international recommendations for antenatal care (45, 131).

An alternative to screening is to supplement all pregnant women and the WHO recommends daily oral iron and folic acid supplementation with 30–60 mg of elemental iron for pregnant women within the context of routine antenatal care, to prevent maternal anaemia, puerperal sepsis, low birth weight and preterm birth (1). The most recent Cochrane meta-analysis supports that daily iron supplements reduces the risk of maternal anemia and ID but states that effects on other health outcomes are less clear (132). However, general supplementation of all pregnant women is not currently recommended in the Nordic countries.

### Vegetarian diets and meat substitutes

Vegetarians have significantly lower iron stores than non-vegetarians (133). This is probably due to a combination of difficulty of reaching the RI and a lower iron bioavailability. The RI 15 mg/day is set for 15% absorption. Vegetarian diets probably have lower bioavailability (5–10%), making it even more important to achieve recommended dietary iron intakes, as well as considering dietary enhancers and inhibitors of iron absorption. A well-composed vegetarian diet including wholegrains, legumes, nuts and seeds, vegetables, fruits, and berries has the potential to secure an adequate iron supply.

Meat-substitutes, based on soybeans and wholegrains, and legumes not only provide iron to the meal but also contain large amounts of phytate, which inhibits iron absorption (134, 135). Furthermore, the nutritional quality of meat-substitutes shows large variation; salt and saturated fat were high in certain products, while other products were more in line with nutritional recommendations (134, 136). As meat substitutes are becoming more available and affordable, it is crucial to address challenges regarding nutritive content and bioavailability. When meals are climate adapted these products could also be used as meat-extenders.

### Upper level of iron intake

Ingestion of an acute overdose of a pharmaceutical iron preparation (20 mg/kg or more of iron) causes mucosal erosion in the stomach and intestine, leading to nausea, abdominal pain, vomiting and diarrhea (137). Higher doses > 20–60 mg/kg/day lead to systemic iron overload and can result in gastrointestinal bleeding, shock, metabolic acidosis, and acute liver failure (137, 105, 122). Iron poisoning can be lethal so iron supplements should always be kept out of reach of children (138). However, acute iron intoxication is not considered in setting an upper level (UL) for diet (105).

Because iron absorption is homeostatically regulated, the risk of dietary iron overload is mainly limited to individuals with hereditary disorders of iron metabolism, especially hemochromatosis, which is a relatively common disorder in the Nordic countries with a reported frequency of homozygosity for the C282Y mutation ranging from 0.20 to 0.75% (139, 140). There is no evidence that heterozygotes for hemochromatosis are at an increased risk of iron overload. Thus, the NNR DRVs and UL for the general population do not apply to patients with this relatively uncommon condition.

The EFSA has not set any UL for iron (105). The Institute of medicine (IOM) (currently the National Academy of Medicine) has set an UL of 40 mg/day for infants and children and 45 mg/day for adolescents and adults (122).

Epidemiological data on the association between iron and the risk of cardiovascular disease, cancer, or diabetes do not permit the establishment of any dose-response relationships with dietary iron. Consequently, a quantitative UL for iron intake cannot be set on this basis, and it is not possible to directly derive a limit based on liver fibrosis or increased hepatic iron and ferritin concentrations. However, it seems clear that a ferritin level above 300 mg/L, which is often referred to as ‘biochemical iron overload’ when caused by increased iron stores, is associated with an increased risk of slight liver fibrosis. Based on homeostatic control of iron absorption and the risk of biochemical iron overload, the UL might occur at intake levels between 10 and 30 mg/day of additional iron over and above typical dietary intakes. A regular intake of 60 mg/day in a fertile woman has been calculated to lead to biochemical iron overload, and a quantitative UL for iron intake in addition to habitual dietary iron is set to 10 mg non-heme iron per day in order to avoid such overload. Based on the given evidence, the UL for total iron intake is 60 mg/day.

Under physiological conditions, iron status is almost exclusively regulated by adaptation of intestinal iron absorption to demand and this process is well described for both deficiency and supply via food. Several studies indicate that this regulation operates up to the level of an additional 10–15 mg iron/day (11, 124, 141, 142). However, Fleming and co-workers found that an additional iron intake of > 30 mg iron per day was associated with an increased risk of high iron stores that are defined as plasma-ferritin > 300 mg/L or > 200 mg/L in elderly men and women, respectively (143). Thus, the homeostatic regulation of iron absorption in elderly people seems able to prevent iron overload at a total iron intake of 17.5–25 mg/day (10 mg/day habitual dietary intake + 7.5–15 mg/day), but not at a total intake of 40 mg/day (10 mg/day habitual dietary intake + 30 mg/day). Theoretical calculations of prolonged intake of pharmaceutical iron and ferritin levels showed that ingestion of an extra 60 mg iron/day over 5 years or more would risk building up excessive iron stores in a fertile non-pregnant woman with a body weight of 63 kg (144). Long-term effects of high iron (12 mg/L) infant formula from 6 to12 months of age in healthy, non-anemic infants were found in a follow-up at 10 years of age as lower scores for visual-motor integration (145). This negative effect seemed to be limited to those infants who were initially iron-replete but suggests possible adverse effects of excessive iron intake during late infancy. NNR 5 set the upper limit for iron for non-pregnant adults at 60 mg/day. It is not possible to set an UL for infants, but infant formulas should not provide more than 8 mg/L (42).

Although it is not possible to establish a cause-effect relationship between iron and diseases, it seems prudent at least in sub-populations such as adult males, post-menopausal women, and heterozygotes for hemochromatosis to avoid an intake of iron above the current recommendation, which already provides for the highest need.

Studies indicate possible adverse effects of high iron intake during late infancy. Infant formulas, therefore, should not provide more than 8 mg/L of iron.

Maintaining an iron intake below the UL would also protect against the local intestinal toxicity that is a side effect of therapeutic iron. The lower dose level of iron associated with such acute side effects seems to be in the range of 50–60 mg/day.

The UL and intake advice do not apply to individuals receiving iron prophylaxis and pharmaceutical iron preparations under medical supervision, such as pregnant women (for whom a supplement should be considered in the amount of 40 mg/day from week 18–20 of gestation) and low birth weight infants.

### Data gaps for future research

- There is an urgent need for randomized, controlled trials of diets with different amounts of bioavailable iron, in settings relevant for the Nordic populations. This is especially important for infants and toddlers at 6–36 months, adolescent girls, vegetarians and vegans. Important outcomes to include are ID anemia, as well as long term neurodevelopmental outcomes in children.
- Effective screening instruments for hypermenorrhea should be identified and implemented in adolescent girls as well as older, menstruating women.
- Climate adapted meals including more vegetarian eating could affect groups with high iron needs.
- There is a need of studies of the amount of phytate in whole grains, nuts and seeds, legumes and meat-substitutes, using standardized analysis methods.
- Meat-substitutes and meat replacement products with improved nutritional quality and iron bioavailability should be developed.

## Appendix 1: Search strategy

iron [Mesh] AND (supplement* OR diet* OR nutrition* OR food) AND human

*Filters applied: Randomized Controlled Trial, Systematic Review, in the last 10 years (time period May 2011 to May 2021)*

iron”[MeSH Terms] AND (“supplement*”[All Fields] OR “diet*”[All Fields] OR “nutrition*”[All Fields] OR (“food”[MeSH Terms] OR “food”[All Fields])) AND (“human s”[All Fields] OR “humans”[MeSH Terms] OR “humans”[All Fields] OR “human”[All Fields])) AND ((y_10[Filter]) AND (randomizedcontrolledtrial[Filter] OR systematicreview[Filter]

yielded 535 results with 234 remaining after manual exclusion of papers not relevant for the NNR.

Additional literature search was performed based on references in the selected papers as well as reference papers regarding iron requirements. Also, some more recent publications from 2021–2022 were added.
